# Crystal structure of the α-ketoglutarate-dependent non-heme iron oxygenase CmnC in capreomycin biosynthesis and its engineering to catalyze hydroxylation of the substrate enantiomer

**DOI:** 10.3389/fchem.2022.1001311

**Published:** 2022-09-13

**Authors:** Yu-Hsuan Hsiao, Szu-Jo Huang, En-Chi Lin, Po-Yun Hsiao, Shu-Ing Toh, I-Hsuan Chen, Zhengren Xu, Yu-Pei Lin, Hsueh-Ju Liu, Chin-Yuan Chang

**Affiliations:** ^1^ Department of Biological Science and Technology, National Yang Ming Chiao Tung University, Hsinchu, Taiwan; ^2^ Institute of Molecular Medicine and Bioengineering, National Yang Ming Chiao Tung University, Hsinchu, Taiwan; ^3^ State Key Laboratory of Natural and Biomimetic Drugs, School of Pharmaceutical Science, Peking University, Beijing, China; ^4^ Department of Applied Chemistry, National Yang Ming Chiao Tung University, Hsinchu, Taiwan; ^5^ Center for Intelligent Drug Systems and Smart Bio-devices, National Yang Ming Chiao Tung University, Hsinchu, Taiwan; ^6^ Department of Biomedical Science and Environment Biology, Kaohsiung Medical University, Kaohsiung, Taiwan

**Keywords:** capreomycin, non-heme iron oxygenase, hydroxylation, engineering, D-Arg

## Abstract

CmnC is an α-ketoglutarate (α-KG)-dependent non-heme iron oxygenase involved in the formation of the l-capreomycidine (l-Cap) moiety in capreomycin (CMN) biosynthesis. CmnC and its homologues, VioC in viomycin (VIO) biosynthesis and OrfP in streptothricin (STT) biosynthesis, catalyze hydroxylation of l-Arg to form β-hydroxy l-Arg (CmnC and VioC) or β,γ-dihydroxy l-Arg (OrfP). In this study, a combination of biochemical characterization and structural determination was performed to understand the substrate binding environment and substrate specificity of CmnC. Interestingly, despite having a high conservation of the substrate binding environment among CmnC, VioC, and OrfP, only OrfP can hydroxylate the substrate enantiomer d-Arg. Superposition of the structures of CmnC, VioC, and OrfP revealed a similar folds and overall structures. The active site residues of CmnC, VioC, and OrfP are almost conserved; however Leu136, Ser138, and Asp249 around the substrate binding pocket in CmnC are replaced by Gln, Gly, and Tyr in OrfP, respectively. These residues may play important roles for the substrate binding. The mutagenesis analysis revealed that the triple mutant CmnC^L136Q,S138G,D249Y^ switches the substrate stereoselectivity from l-Arg to d-Arg with ∼6% relative activity. The crystal structure of CmnC^L136Q,S138G,D249Y^ in complex with d-Arg revealed that the substrate loses partial interactions and adopts a different orientation in the binding site. This study provides insights into the enzyme engineering to α-KG non-heme iron oxygenases for adjustment to the substrate stereoselectivity and development of biocatalysts.

## Introduction

α-Ketoglutarate (α-KG)-dependent non-heme iron oxygenases catalyze diverse reactions involved in many natural product biosynthesis (Gao et al., 2018; Nakamura et al., 2018). These enzymes share initial steps of catalysis and a general enzyme mechanism (Martinez and Hausinger, 2015). The active site dioxygen supersedes the water molecule to form a Fe^II^–superoxide anion or a Fe^III^–superoxide. The superoxide then attacks the carbonyl carbon of α-KG, resulting in the decarboxylation product succinic acid and the reactive Fe^IV^–oxo species. The reactive Fe^IV^–oxo typically undergoes diverse reactions through radical processes. For example, Fe^IV^–oxo abstracts a H atom (H·) from the carbon atom of the substrate to form a Fe^III^–OH cofactor and the corresponding carbon radical (C·). The hydroxylation reaction is subsequently performed *via* the rebound recombination between the carbon radical and the iron-bound hydroxyl group (Hausinger, 2004; Yin and Zabriskie, 2004; You et al., 2007; Chang et al., 2014). In addition to hydroxylation, epoxidation, desaturation, cyclization, and halogenation are followed a similar catalytic mechanism (Blasiak et al., 2006; Gao et al., 2018; Nakamura et al., 2018; Li et al., 2020). Intriguingly, some enzymes in this versatile family of oxygenases catalyze multiple reactions. For example, clavaminate synthase (CAS) catalyzes sequential hydroxylation, cyclization, and desaturation reactions in clavulanic acid biosynthesis (Janc et al., 1995; Zhang et al., 2000); AusE and PrhA catalyze desaturation reactions, followed by intramolecular rearrangement to condense/expand the rings in austinol and paraherquonin biosynthesis, respectively (Nakashima et al., 2018).

**FIGURE 1:**
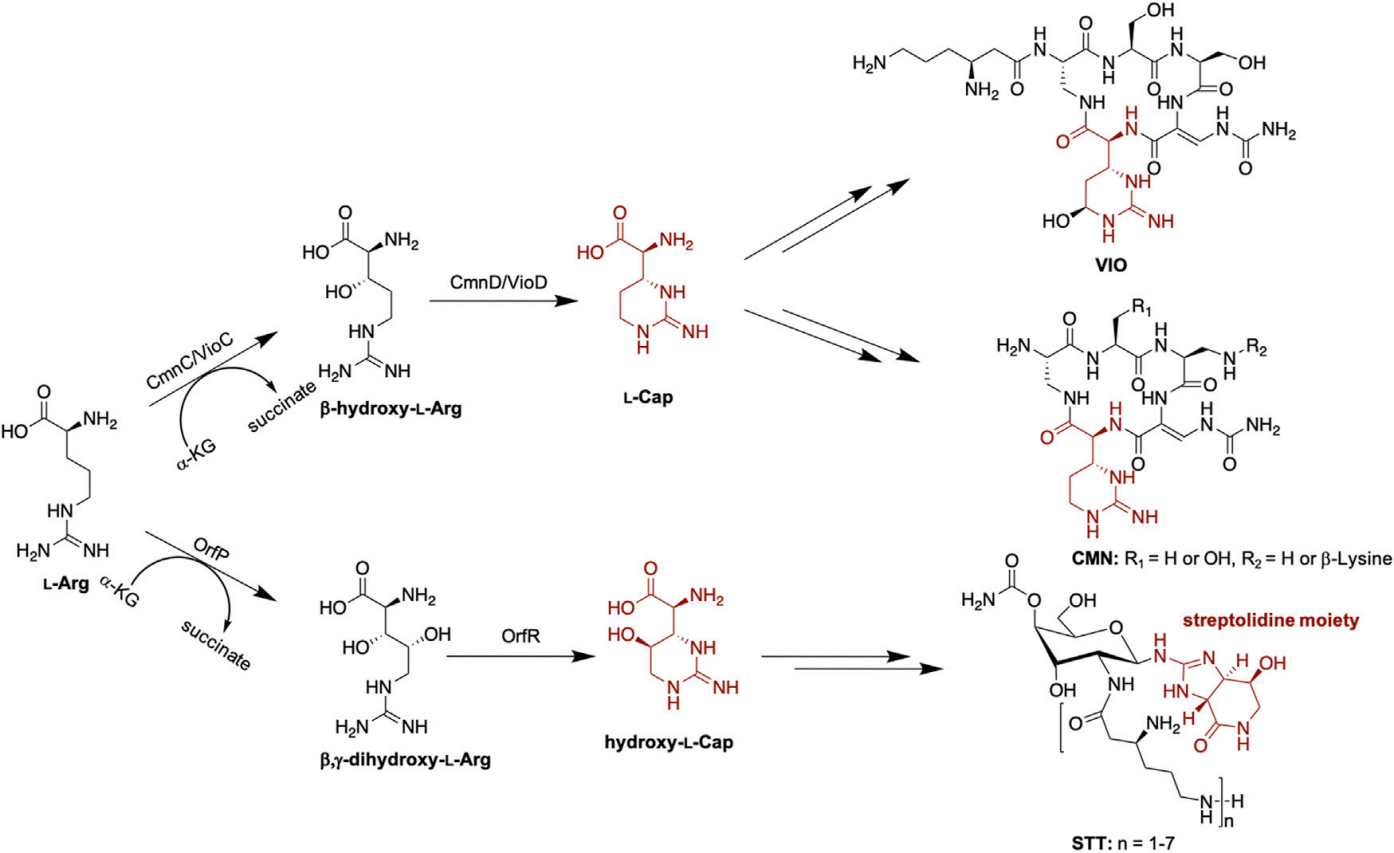
Proposed biosynthesis of l-Cap in VIO, CMN, and STT biosynthetic pathways. Hydroxy-l-Cap is an intermediate to form the streptolidine moiety in STT biosynthesis.

In addition to the 20 standard amino acids, non-proteinogenic amino acids expand the structural diversity of non-ribosomal peptides (NRPs). α-KG-dependent non-heme iron oxygenases were reported to participate in the biosynthesis of non-proteinogenic amino acids, incorporated into the non-ribosomal peptide synthetase (NRPS) machineries. VioC and OrfP are α-KG-dependent non-heme iron oxygenases involved in the first step of the non-proteinogenic amino acids capreomycidine (Cap) and hydroxy-Cap formation in viomycin (VIO) and streptothricin (STT) biosynthesis, respectively (Figure 1) (Ju et al., 2004; Yin and Zabriskie, 2004; Chang et al., 2014). VioC and OrfP catalyze hydroxylation and double hydroxylation of l-Arg to form β-hydroxy l-Arg and β,γ-dihydroxy l-Arg, respectively. VioD and OrfR, the PLP-dependent enzymes, subsequently undergo dehydration and cyclization of the hydroxylated products from the last step and afford the six-membered ring product Cap and hydroxy-Cap (Figure 1) (Ju et al., 2004; Yin et al., 2004). The crystal structures of VioC and OrfP revealed that they share a similar fold and active site environment (Helmetag et al., 2009; Chang et al., 2014). The catalytic iron is coordinated by the conserved 2-His-1-Glu facial triad within the HXD/EX_n_H Fe binding motif. The substrate l-Arg is bound in the active site pocket, where the conserved residues, Arg and Asp, at the opposite faces stabilize the carboxylate and guanidino groups of the substrate through electrostatic interactions, respectively. Intriguingly, despite high sequence and structure similarity between VioC and OrfP, they show different substrate specificity and reaction products (Helmetag et al., 2009; Chang et al., 2014; Dunham et al., 2018a). As mentioned previously, OrfP catalyzes double hydroxylation of l-Arg at β and γ positions (Chang et al., 2014), while VioC catalyzes single hydroxylation at β position (Yin and Zabriskie, 2004). Furthermore, OrfP accepts the substrate enantiomer d-Arg and affords the hydroxylated product. VioC is unable to catalyze hydroxylation of d-Arg; however, it catalyzes an efficient oxidative deamination of d-Arg to form the corresponding 2-oxo-acid (Helmetag et al., 2009; Dunham et al., 2018b).

Capreomycin (CMN) and VIO are structural analogs (Figure 1). CMN and VIO belong to the tuberactinomycin family of antibiotics and are among the most effective antibiotics against tuberculosis. The biosynthetic gene clusters (BGCs) of CMN and VIO have been identified and sequenced (Thomas et al., 2003; Felnagle et al., 2007; Barkei et al., 2009) and the function of each gene encoded within each individual BGC has been functionally annotated or characterized (Thomas et al., 2003; Fei et al., 2007; Hsu et al., 2021; Pan et al., 2022). The gene arrangement in both gene clusters are similar, implicating a similar biosynthetic pathway. CmnC, the VioC homologue, was proposed to catalyze β-hydroxylation of l-Arg involved in Cap biosynthesis (Figure 1). Herein, we investigated the substrate specificity of CmnC with a group of l-Arg analogs, including d-Arg, l-homoarginine, l-canavanine, l-citrulline, l-norarginine, N5-(1-iminoethyl)-l-ornithine, and l-homocitrulline. In addition, the crystal structures of CmnC were determined for the comparative structural study with VioC and OrfP. By structure-based enzyme engineering, the triple mutant CmnC^L136Q,S138G,D249Y^ is capable of accepting the substrate enantiomer d-Arg for hydroxylation.

## Materials and methods

### Gene cloning and site-directed mutagenesis of CmnC

The *cmnC* (GenBank accession ID: ABR67746.1) from *Streptomyces mutabilis* subsp. *capreolus* (ATCC 23892) was amplified from genomic DNA by polymerase chain reaction (PCR). The amplified gene was subcloned into expression vector pET28a. For the triple mutant CmnC^L136Q,S138G,D249Y^, the plasmid was constructed by the QuikChange site-directed mutagenesis method. The primers used for the wild-type and the triple mutant CmnC are shown in Supplementary Table S1. All the plasmids used in this study were verified by DNA sequencing. Each of the wild-type and the mutant constructs was transformed into *Escherichia coli* BL21 (DE3) for gene expression and protein production.

### Expression, production, and purification of CmnC and its triple mutant

Both recombinant CmnC and its triple mutant were produced by a general procedure (Chang et al., 2014). For gene expression and protein production, 1 L LB medium was inoculated with 3 ml of an overnight *E. coli* BL21 (DE3) culture grown in LB medium containing 100 mg/ml kanamycin, induced by 300 μl 1 M IPTG at an OD_600_ of 0.5. Cells were grown for an additional 12 h at 18°C and were harvested by centrifugation at 6,000 rpm for 30 min at 4°C. For protein purification, the cells harvested by centrifugation were subsequently resuspended in lysis buffer containing 500 mM NaCl, and 20 mM Tris at pH 8.0. The cells were disrupted by sonication. The cell pellets after sonication were removed by centrifugation at 12,000 rpm. Both CmnC and the triple mutant were purified using Ni-NTA affinity chromatography [HisTrap FF (GE Healthcare Life Science)]. All chromatography were performed by NGC Chromatography Systems (Bio-Rad). The purities of both proteins were justified by SDS-PAGE. The pure CmnC and the triple mutant were concentrated using Amicon Ultra-15 concentrator (Merck) in 100 mM NaCl and 20 mM Tris buffer at pH 8.0 for the following enzyme activity assay and protein crystallization. Protein concentrations were determined from the absorbance at 595 nm using Micro BCA protein Assay Kit (Thermo Scientific).

### Activity assay of CmnC and its triple mutant

The CmnC activity assay was followed by the procedures carried out in OrfP (Chang et al., 2014). The hydroxylation reaction was performed in a final volume of 200 μL containing a given substrate (1 mM), α-KG (1 mM), FeCl_2_ (0.05 mM), ascorbic acid (0.2 mM) and purified enzyme (5 μM) in a MOPS buffer (50 mM, pH 7.0). The reaction was incubated at 26°C for 5 h. Protein was precipitated with HCl, and the supernatant was collected for dansyl chloride (DNS-Cl) derivatization. For dansylation, the reaction mixture (100 μl) was added sequentially with solutions of 80 mM Li_2_CO_3_ (100 μl, in water) and 3 mM DNS-Cl (100 μl, in acetonitrile). The reaction mixture was then filtered and subjected to HPLC-ESI-LTQ analysis (Phenomenex Luna-C18 column, 3 μm; 4.6 × 250 mm, Agilent 1200 Series HPLC interface with an ESI source coupled to a Thermo-Finnigan LTQ-XL ion trap spectrometer) at a flow rate of 1 ml/min with three-stage elution gradient (% = A/B × 100%, A: deionized water with 0.1% trifluoroacetic acid, B: acetonitrile with 0.1% trifluoroacetic acid): 98% for 5 min, a linear gradient to 70% in 27 mins, a second linear gradient to 2% in 5 min. The UV wavelength was set at 254 nm.

### CmnC product characterization

The CmnC product, compound **1**, was dansylated and collected by HPLC. The separation condition is the same as that in enzyme activity assay. NMR analysis was carried out on a Varian Vnmrs-600 spectrometer. The NMR spectra were referenced to residual protonated solvent for ^1^H NMR (4.75 ppm for compounds in D_2_O). The compound was dissolved in D_2_O, and spectra were recorded at 25°C. NMR peaks are listed below; all NMR spectra can be found in Supplementary Figure S1.

Dansylated compound **1** (CmnC product): ^1^H-NMR (600 MHz, D_2_O): 8.68 (d, *J* = 9.0 Hz, 1H), 8.31 (d, *J* = 8.4 Hz, 1H), 8.28 (d, *J* = 7.2 Hz, 1H), 7.93 (d, *J* = 8.4 Hz, 1H), 7.76 (m, 2H), 3.73 (m, 1H), 3.65 (d, *J* = 6.6 Hz, 1H), 3.36 (s, 6H), 3.07 (m, 2H), 1.60 (m, 1H), 1.49 (m, 1H) ppm. ^13^C NMR (150 MHz, D_2_O): 172.9, 156.6, 138.9, 134.7, 131.0, 128.7, 127.9, 126.6, 126.3, 126.1, 125.6, 119.2, 68.7, 61.4, 46.6, 37.5, 30.9 ppm. The ^1^H-^1^H Correlation Spectroscopy (COSY) and ^1^H-^13^C Heteronuclear Single Quantum Coherence (HSQC) spectra of this compound are shown in Supplementary Figure S1.

### Determination of the size of CmnC by size-exclusion chromatography

Size-exclusion chromatography was carried out using a Superdex^TM^ 200 Increase 10/300 GL (Cytiva) for CmnC with by NGC Chromatography Systems (Bio-Rad) at 4°C and 1 ml of sample loaded per run. The column was calibrated with Gel Filtration Standard (Bio-Rad) and developed with the elusion buffer (100 mM NaCl and 20 mM Tris, pH 8.0) at a flow rate of 1 ml/min.

### Crystallization and data collection of CmnC

CmnC^apo^ and CmnC^L136Q,S138G,D249Y^ were crystallized using the sitting drop vapor-diffusion method. CmnC and CmnC^L136Q,S138G,D249Y^ were concentrated to 10 mg/ml and were crystallized in a screen condition: 200 mM ammonium acetate, 24% v/v polyethylene glycol 400, and 0.1 M sodium citrate tribasic dihydrate, pH 5.5, at 20°C. The CmnC^α-kg^ complex and CmnC^L136Q,S138G,D249Y^ in complex with α-KG (CmnC^triple,α-kg^) were obtained by soaking 3 mM α-KG and 0.1 mM FeCl_2_ in the crystals for 12 h. CmnC^L136Q,S138G,D249Y^ in complex with d-Arg (CmnC^triple,d−Arg^) was obtained by soaking 20 mM d-Arg in the crystals for 12 h. The CmnC^l−Arg^, CmnC^l-hArg^ and CmnC^l−Arg,α-kg^ complexes were obtained by soaking 3 mM L-Arg/, l-hArg and l-Arg/α-KG as well as 0.1 mM FeCl_2_, respectively, in the CnmC crystals using the hanging drop vapor-diffusion method in a screen condition: 1 M potassium sodium tartrate and 100 mM MES/sodium hydroxide, pH 6.0. The diffraction data were collected at National Synchrotron Radiation Research Center (NSRRC, Taiwan) on the TPS 05A beamline for CmnC^apo^ and CmnC^triple,α-kg^ using a wavelength of 1.0000 Å with the Rayonix MX300HS CCD detector, TLS 13B1 beamline for CmnC^α-kg^, CmnC^l−Arg^ CmnC^l-hArg^, and CmnC^triple,d−Arg^ using a wavelength of 0.9732 Å with the ADSC Quantum-315r CCD detector, and TLS 15A1 beamline for CmnC^l−Arg,α-kg^ using a wavelength of 0.9732 Å with the Rayonix MX300HE CCD detector. All data were indexed and scaled with HKL 2000 (Otwinowski and Minor, 1997).

### Structure determination and refinement

The structure of CmnC^apo^ was solved by the molecular replacement method of MOLREP (Vagin and Teplyakov, 1997) using the structure of VioC (PDB entry 2WBO) (Helmetag et al., 2009) as a search model. Extensive manual model building was performed using COOT (Emsley and Cowtan, 2004; Emsley et al., 2010). The models were refined with REFMAC (Murshudov et al., 1997). The structure of CmnC^apo^ was then used as the phase model for determination of the complex structures, CmnC^α-kg^, CmnC^l−Arg^, CmnC^l-hArg^ and CmnC^l−Arg,α-kg^, CmnC^triple,α-kg^, and CmnC^triple,d−Arg^. The structural refinement for all the complex structures were carried out by the same procedures of CmnC^apo^. The atomic coordinates and structure factors of CmnC^apo^, CmnC^α-kg^, CmnC^l−Arg^, CmnC^l-hArg^ and CmnC^l−Arg,α-kg^, CmnC^triple,α-kg^, and CmnC^triple,d−Arg^ have been deposited in the Protein Data Bank with the accession code 7VGL, 7VGN, 7Y5F, 7Y5I, 7Y5P, 7YHE, and 7YW9, respectively. Data processing and refinement statistics are summarized in Supplementary Table S2.

## Results and discussion

### Characterization of CmnC revealing a β-hydroxylase like VioC

**FIGURE 2:**
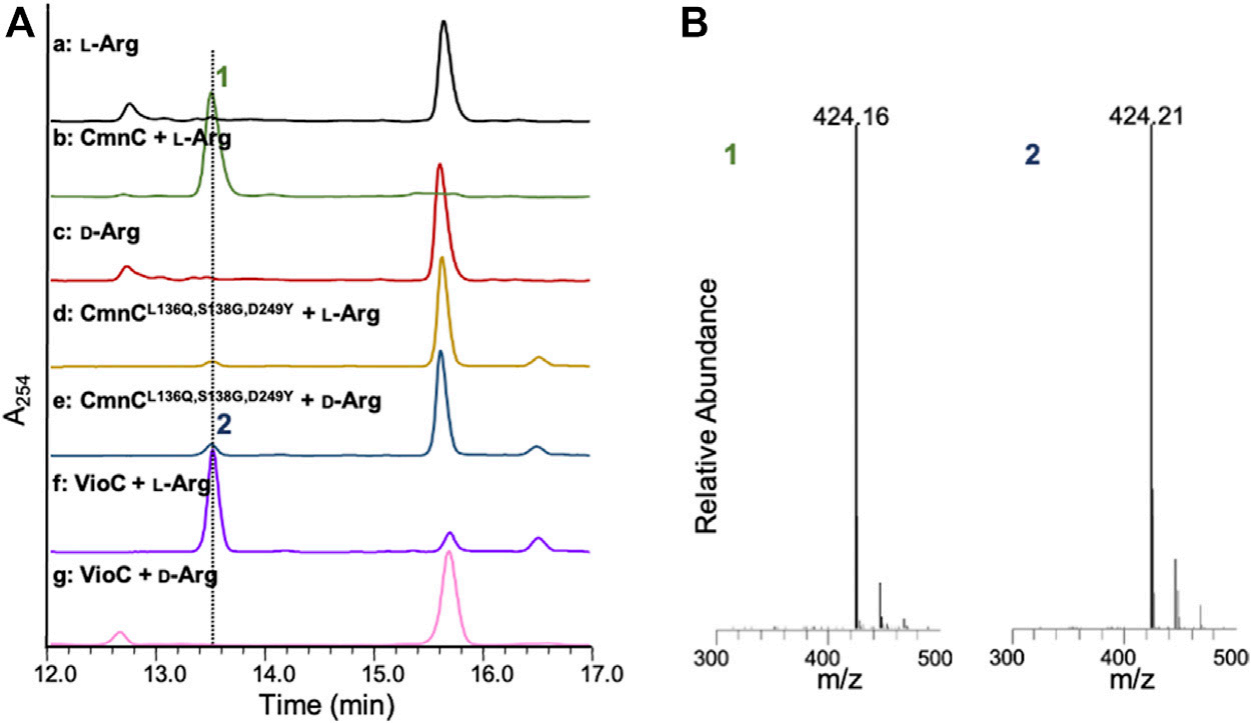
LC-MS analysis of the CmnC and VioC reactions. **(A)** HPLC traces of the substrate l-Arg and the enantiomer d-Arg (red) and reactions in the presence of CmnC with l-Arg (green), the triple mutant CmnC^L136Q,S138G,D249Y^ with l-Arg (yellow) or d-Arg (blue), and VioC with l-Arg (purple) or d-Arg (pink). **(B)** Mass spectrometric analysis of the reaction products. Compound **1** and **2** are the reaction products of CmnC against l-Arg and CmnC^L136Q,S138G,D249Y^ against d-Arg, respectively. All samples were dansylated before LC-MS analysis.

CmnC shares 61.7% amino acid sequence identity with VioC. Like VioC, CmnC was proposed to catalyze β-hydroxylation of l-Arg in Cap biosynthesis (Figure 1). To confirm the function of CmnC, we amplified the gene *cmnC* from the CMN producing strain *Saccharothrix mutabilis* subsp. *capreolus* and was subsequently subcloned into expression vector pET28a for gene expression. The CmnC recombinant protein was produced in *E. coli* and purified for enzyme activity assay (Supplementary Figure S2). The enzyme reaction was performed in a typical non-heme iron oxygenase reaction with l-Arg as a substrate (Helmetag et al., 2009; Chang et al., 2014; Dunham et al., 2018a). A new peak was observed in accompanied with consumption of the substrate l-Arg on the LC/MS trace (Figure 2). We also produced and purified the VioC recombinant protein for enzyme activity assay, which has been done in previous study (Yin and Zabriskie, 2004). The results revealed that the retention time of the new peak from the CmnC reaction is the same as the VioC product, β-hydroxy l-Arg (**1**) (Figure 2A). The mass spectrometry analysis suggested that the CmnC product is hydroxylated l-Arg. The mass units of the CmnC product increased by 16 a.m.u. compared to that of the substrate l-Arg (Figure 2B; Supplementary Figure S3). The CmnC product (**1**) was then separated, collected, and subjected to NMR analysis (Supplementary Figure S1). The compound was determined to be β-hydroxy l-Arg. The stereochemistry of the CmnC product was proposed to be the same as that of the VioC product, 3*S*-hydroxy l-Arg, due to the same biosynthetic pathway and stereochemistry of the l-Cap moiety in CMN and VIO (Figure 1) (Yin and Zabriskie, 2004; Felnagle et al., 2007; Helmetag et al., 2009). In addition, the crystal structures of CmnC and VioC revealed that their active sites are almost identical (crystal structures will be discussed below), implicating the same stereochemistry of the products. Therefore, the stereochemistry of the hydroxyl group of the CmnC product (**1**) was proposed to be the *S* configuration.

### CmnC revealed broad substrate specificity for hydroxylation of l-arg analogs

In previous studies, VioC and OrfP were reported that they can hydroxylate not only l-Arg but also its analogs. VioC catalyzes hydroxylation of l-homoargnine (l-hArg) and l-canavanine (l-Cana) (Helmetag et al., 2009). Interestingly, in addition to the hydroxylation reaction, VioC catalyzes C–C and C–N desaturation of l-hArg and d-Arg (Dunham et al., 2018a; Dunham et al., 2018b), respectively. On the other hand, OrfP catalyzes hydroxylation or double hydroxylation of l-hArg and l-canavanine. Intriguingly, OrfP is capable of hydroxylating the substrate enantiomer, d-Arg, with high activity (Chang et al., 2014). To understand the substrate specificity of CmnC, the l-Arg analogs, d-Arg, l-hArg, l-Cana, l-norarginine (l-nArg), N5-(1-iminoethyl)-l-ornithine (l-NIO), l-citrulline (l-Citru), and l-homocitrulline (l-hCitru), were used as substrates for enzyme activity assay (Supplementary Figure S4). The recombinant proteins VioC and OrfP were also produced and purified for enzyme activity assay with the l-Arg analogs in the same condition (Supplementary Figures S4–S11). The relative activity was summarized in Table 1. The results revealed that CmnC, VioC and OrfP show a similar substrate specificity. Like VioC and OrfP, CmnC accepts a slightly modified side chain of the substrates. CmnC can also accept l-hArg and l-Cana as the substrates for hydroxylation; however, CmnC shows significantly higher relative activity with l-nArg and l-NIO than that of VioC and OrfP (Table 1). The crystal structures of CmnC and VioC revealed the conserved residues in substrate binding (crystal structures will be discussed below); however, subtle differences in the orientation of these binding residues were found between CmnC and VioC (Figure 5B). The differences might affect the substrate binding and enzyme catalysis, resulting in different relative activities between CmnC and VioC. All CmnC, VioC, and OrfP show inactive to l-Citru and l-hCitru. Most notably, only OrfP shows ability for d-Arg hydroxylation; both CmnC and VioC are incapable of hydroxylating to d-Arg (Table 1).

**TABLE 1:** Relative activities of CmnC, VioC, and OrfP against l-Arg and its analogs.

Enzyme substrate	CmnC	VioC	OrfP
l-Arg	98.8 ± 0.4%	98.8 ± 1.8%	33.3 ± 0.8%
d-Arg	N.D.	N.D.	37.4 ± 0.7%
l-hArg	44.4 ± 2.1%	40.1 ± 2.0%	55.3 ± 0.6%
l-Cana	8.8 ± 2.2%	8.5 ± 1.0%	19.9 ± 1.4%
l-nArg	15.6 ± 0.3%	1.0 ± 0.4%	8.8 ± 0.2%
l-NIO	35.9 ± 1.2%	3.3 ± 0.5%	9.6 ± 0.6%
l-Citru	<1.0%	<1.0%	<1.0%
l-hCitru	N.D.	N.D.	N.D.

### The structure of CmnC is similar to that of VioC, revealing conserved active site residues for substrate binding and catalysis

**FIGURE 3:**
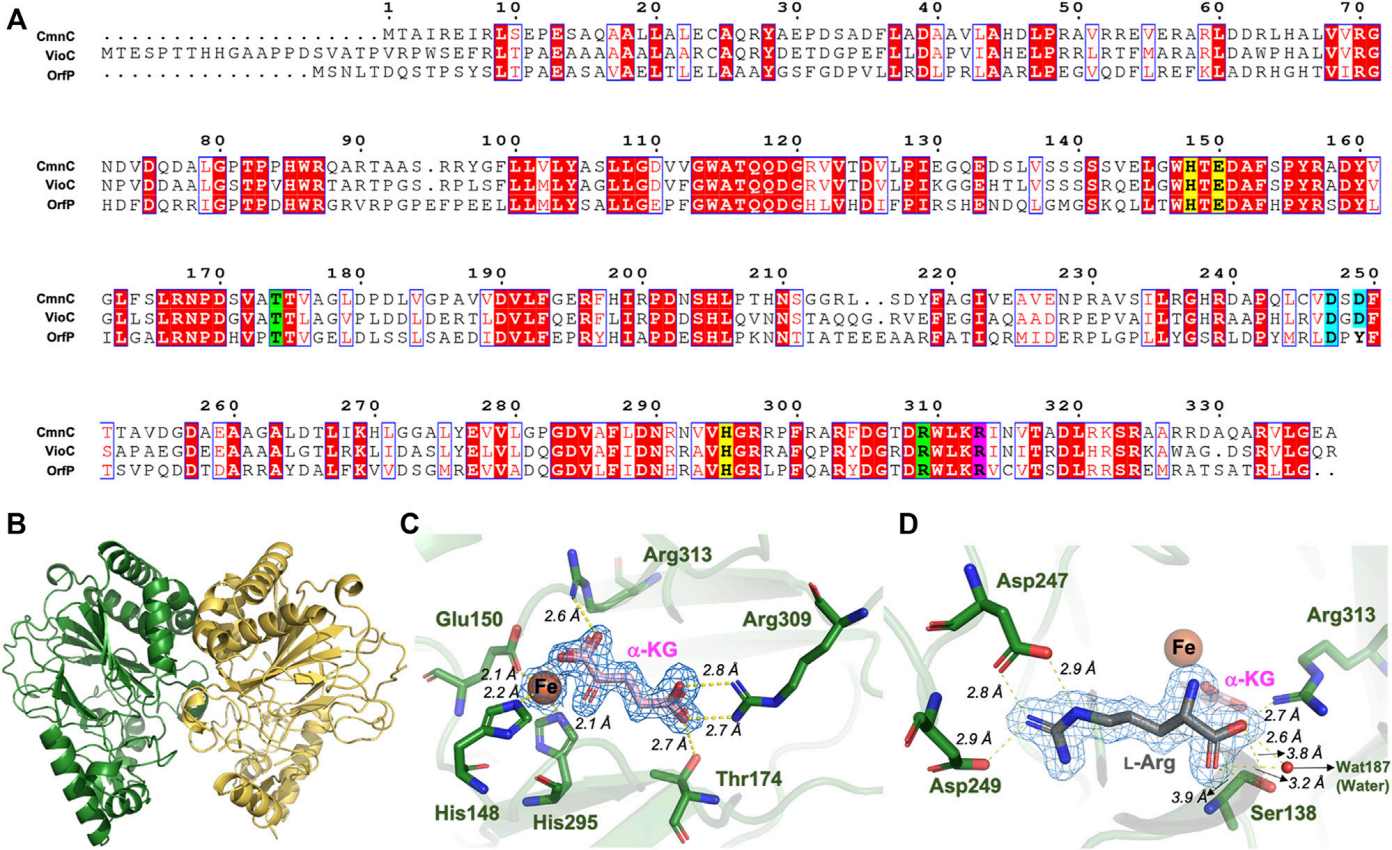
Sequence alignment and crystal structure of CmnC. **(A)** Sequence alignment of CmnC with VioC and OrfP. Aligned residues are colored on the bases of the level of conservation (red background shows strict identity, red character for similarity, and blue frame for similarity across group). The 2-His-1-Glu facial triad is highlighted in yellow background. Residues involved in α-KG and l-Arg binding are highlighted in green and cyan background, respectively. Residues involved in both α-KG and l-Arg binding are highlighted in purple background. The figure was generated by ESPript 3.0. **(B)** Overall structure of CmnC dimer. Two polypeptide chains are colored green and yellow, respectively. **(C)** Local view of the Fe and α-KG binding site. **(D)** Local view of the l-Arg binding site. The electrostatics and hydrogen bond interactions are depicted as yellow dotted lines. The composite (2*mF*_*o*_–*F*_*c*_) omit maps contoured at 1.0 σ in **(C,D)** are colored blue.

CmnC shares high sequence identities, 61.7 and 42.9%, with VioC and OrfP, respectively (Felnagle et al., 2007; Chang et al., 2014) (Figure 3A). They show similar substrate specificity but different relative activity (Table 1). The crystal structures of VioC and OrfP were determined in previous studies. In this study, we solved the crystal structure of CmnC. Five high-resolution crystal structures of CmnC in free form (CmnC^apo^) and in complex with l-Arg (CmnC^l−Arg^), l-hArg (CmnC^l-hArg^), α-KG (CmnC^α-kg^), and l-Arg/α-KG (CmnC^l−Arg,α-kg^) were solved at resolutions of 1.49–1.83 Å. The complex structures were obtained by socking crystals of CmnC^apo^ with proper concentrations of l-Arg, l-hArg, or α-KG. Each of CmnC structures was indexed with the same orthorhombic space group *I222*, and the CmnC^apo^ structure was determined by the molecular replacement (MR) method using the structure of VioC (PDB entry 2WBO). The structure of CmnC^apo^ was then used as the phase model for determining the CmnC complex structures. Data processing and refinement statistics are summarized in Supplementary Table S2. An asymmetric unit of either the apo form or the complex forms of CmnC contains two polypeptide chains in a dimer form (Figure 3B), which is consistent with the analysis of size-exclusion chromatography (Supplementary Figure S12). The electron density is uninterrupted and traceable from the first residue at the N-terminus all of the way to the end of the C-terminus for both apo and complex forms, with the region between His205 and Ser216 lacking significant density in one polypeptide chain of the CmnC dimer. The extra electron density was identified and unambiguously matched with the soaking components, l-Arg, l-hArg, and α-KG. In addition, a tartrate molecule from the crystallization condition is found in the active site, where it occupies the α-KG binding site and coordinates with the catalytic iron in the complex structures CmnC^l−Arg^ and CmnC^l-hArg^.

**FIGURE 4:**
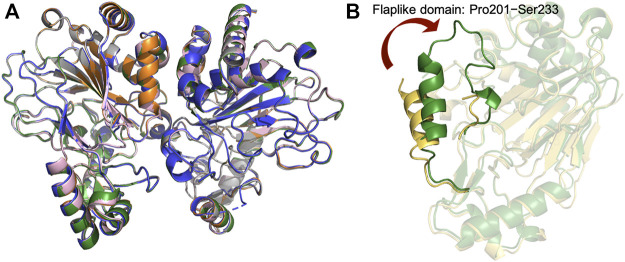
Crystal structures of CmnC in free form and the complex structures. **(A)** Superposition of the structures of CmnC^apo^ (green), CmnC^l−Arg^ (orange), CmnC^l-hArg^ (grey), CmnC^α-kg^ (blue), and CmnC^l−Arg,α-kg^ (pink). **(B)** Superposition of two polypeptide chains from the CmnC^apo^ dimer. The two polypeptide chains are colored green and yellow.

The overall structure of CmnC shares similar folds with canonical non-heme iron oxygenases. The catalytic iron is bound by the 2-His-1-Glu facial triad comprised of His148, Glu150, and His295 (Figure 3C). Thr174, Arg309, and Arg313 interact with the carboxylate groups of α-KG *via* hydrogen bond and electrostatic interactions. The α-ketoacid bidentate group provides additional coordination to the catalytic iron (Figures 3A,C). The substrate, l-Arg, was bound in an amphoteric binding pocket, where Asp247/Asp249 and Arg313/Ser138 at both ends interact with the guanidino and carboxylate groups of the substrate, respectively, *via* electrostatic and hydrogen bond interactions (Figures 3A,D). The previous studies reported that α-KG-dependent non-heme iron oxygenases revealed a substantial conformational change; the open form allows substrates to enter the active site, and the closed form enables the formation of the reactive oxygen species (Chang et al., 2014). The superposition of CmnC^apo^ and the complex structures, CmnC^l−Arg^, CmnC^l-hArg^, CmnC^α-kg^ and CmnC^l−Arg,α-kg^, shows relatively small root-mean-square deviations (rmsds) of 0.105–0.168 Å for Cα atoms, suggesting that CmnC does not undergo a significant conformational change upon binding of l-Arg, l-hArg, and α-kg (Figure 4A). However, a flaplike domain (Pro201–Ser233) adopts two structural modes within two polypeptide chains from the CmnC dimer, implicating that CmnC may undergo conformational change while the reaction proceeds (Figure 4B).

The crystal structure and catalytic mechanism of VioC have been reported (Ju et al., 2004; Yin and Zabriskie, 2004; Helmetag et al., 2009). CmnC retains all the active site residues with VioC, including residues for Fe, α-KG and substrate binding (Figure 3). A canonical mechanism for the hydroxylation reaction was proposed to proceed by CmnC. Next, we focus on the comparative structural study to understand the substrate specificity in CmnC, VioC, and OrfP.

### Sequence and structural comparisons of CmnC with VioC and OrfP implicating key residues for substrate stereoselectivity

**FIGURE 5:**
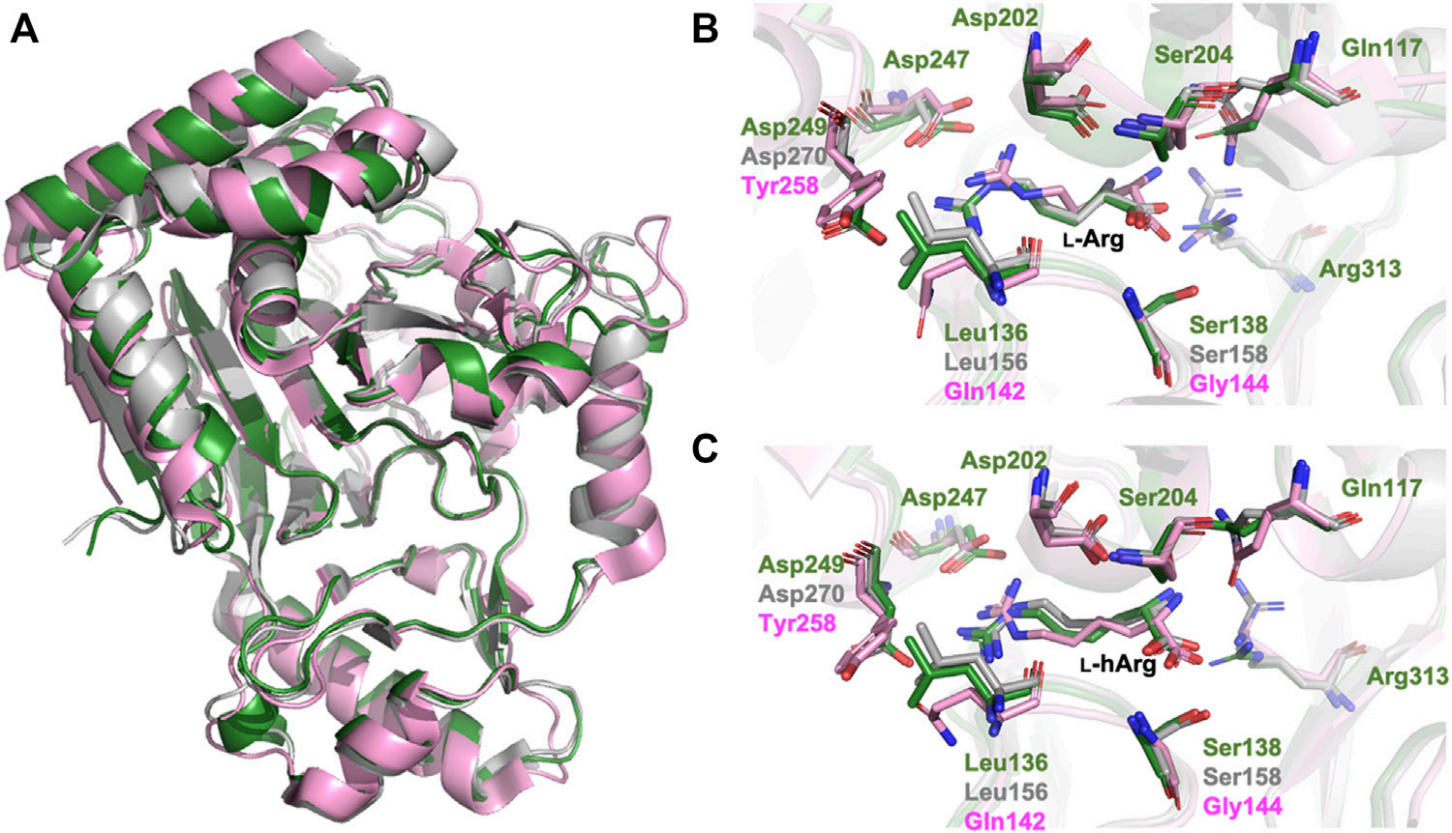
Structural comparison of CmnC, VioC, OrfP. **(A)** Superposition of the overall structures of CmnC, VioC, and OrfP. Local view of the **(B)** l-Arg binding and **(C)** l-hArg binding in CmnC, VioC, and OrfP. CmnC, VioC, and OrfP are colored green, grey, and pink, respectively.

 CmnC, VioC, and OrfP share nearly identical three-dimensional structures with 0.447–0.998 Å rmsds for Cα atoms. The active site residues involved in substrate binding are almost consistent (Figures 3A, 5A); however, only OrfP is capable of catalyzing hydroxylation to the substrate enantiomer d-Arg (Supplementary Figure S5). Structural superposition of CmnC, VioC, and OrfP revealed that three residues, Leu136/Leu156, Ser138/Ser158 and Asp249/Asp270 around the substrate binding site in CmnC/VioC are replaced by Gln142, Gly144 and Tyr257 in OrfP, respectively (Figures 5B,C). These three residues may contribute to affect the substrate binding pocket with slightly differences, resulting in a different substrate orientation in CmnC/VioC and OrfP. Structural comparison for the substrate binding revealed that Ser138/Ser158 and Asp249/Asp270 in CmnC/VioC provide additional electrostatic and hydrogen bond interactions to fix the substrate orientation (Figures 3D, 5B,C). The substrate stereoselectivity was suggested to be restricted by the effect of substrate orientation in CmnC/VioC, which was unable to hydroxylate the substrate enantiomer d-Arg. To assess whether we could control the substrate stereoselectivity of CmnC, we performed site-directed mutagenesis and created a CmnC^L136Q,S138G,D249Y^ triple mutant, of which the three residues around the substrate binding pocket were mutated as the same with that on OrfP. The activity assay revealed that the triple mutant CmnC^L136Q,S138G,D249Y^ almost abolished the hydroxylation activity with ∼3% relative activity to l-Arg; however, it showed ∼6% relative activity to d-Arg (**2**) (Figure 2). The mass spectrometry analysis suggested that the compound **2** is hydroxylated d-Arg, of which the mass units increased by 16 a.m.u. compared to that of d-Arg. The structure-based enzyme engineering changes the substrate stereoselectivity of CmnC that accepts d-Arg for hydroxylation.

**FIGURE 6:**
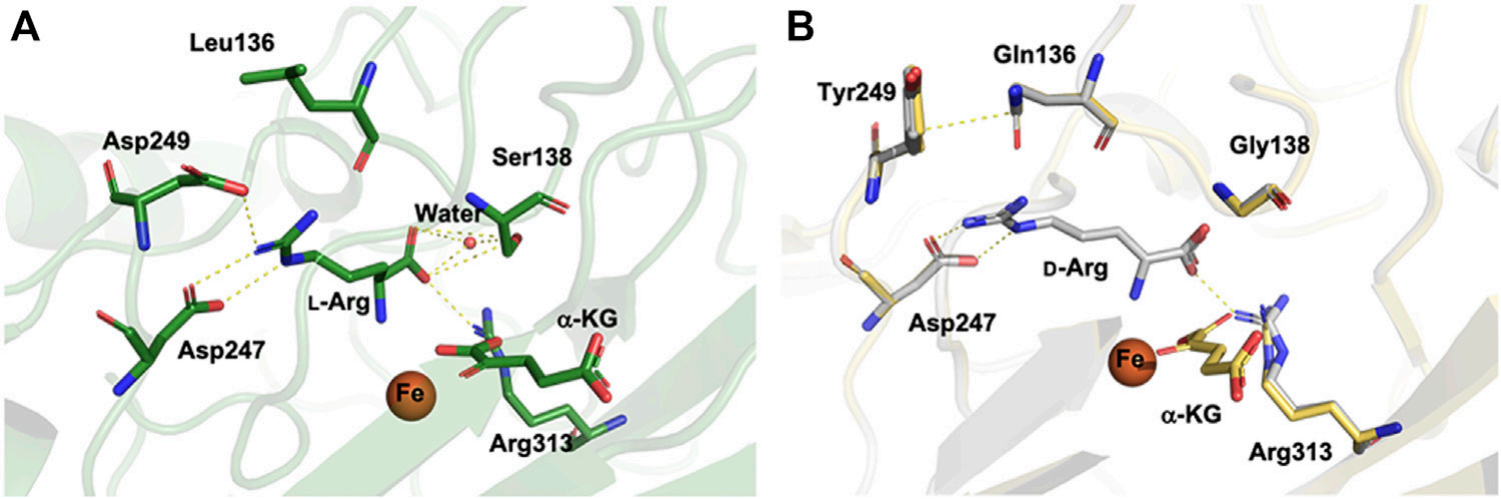
Structural comparison of CmnC^l−Arg,α-kg^ and CmnC^L136Q,S138G,D249Y^. **(A)** Local view of the active sites of CmnC^l−Arg,α-kg^. **(B)** Superposition of the structures and the local view of the active sites of CmnC^triple,α−KG^ (yellow) and CmnC^triple,d−Arg^ (grey). The electrostatics, hydrogen bond, and π–electron interactions are depicted as yellow dotted lines.

To illustrate the molecular detail of the engineered CmnC that switched the substrate stereoselectivity, we solved the crystal structures of CmnC^L136Q,S138G,D249Y^ in complex with α-KG (CmnC^triple,α-kg^) and d-Arg (CmnC^triple,d−Arg^) at resolutions of 1.67 Å and 1.75 Å, respectively. Data processing and refinement statistics are summarized in Supplementary Table S2. The complex structures of the CmnC^L136Q,S138G,D249Y^ triple mutants revealed almost identical active site environment with that of the wild-type CmnC. The residues involved in Fe and α-KG binding show no significant change between CmnC^l−Arg,α-kg^ and CmnC^triple,α-kg^ (Figure 6). For the substrate binding pocket, Asp249 and Ser138 in the wild-type CmnC bind the guanidino group and carboxylate group of the substrate l-Arg, respectively. Furthermore, a water molecule bound by Ser138 mediates the hydrogen bond and help to bind the substrate (Figure 6A). In contrast, the side chains of Gln136 and Tyr249 in the triple mutant CmnC^L136Q,S138G,D249Y^ make close contact through π–electron interactions. In addition, the water molecule bound by Ser138 in the wild-type CmnC is lost in the triple mutant (Figure 6B). The crystal structure of CmnC^triple,d−Arg^ revealed that the substrate d-Arg loses partial interactions and adopts a different orientation in the binding site compared to the substrate l-Arg in the wild-type CmnC. Nevertheless, the relative activity of CmnC^L136Q,S138G,D249Y^ for d-Arg hydroxylation is not as high as the wild-type CmnC for l-Arg hydroxylation, implicating that there are more residues participate in the substrate stereoselectivity.

## Conclusion

 In summary, the function and the substrate specificity of CmnC were investigated. CmnC revealed broad substrate specificity for hydroxylation of l-Arg analogs, l-hArg, l-Cana, l-nArg, and l-NIO, with 8–44% relative activities (Table 1). Furthermore, we determined the crystal structures of CmnC and its triple mutant CmnC^L136Q,S138G,D249Y^, and mapped out the residues that influence the substrate stereoselectivity. The comparative structural study enables us to speculate on the most promising residues for the substrate stereoselectivity in CmnC. The activity assay revealed that the triple mutant CmnC^L136Q,S138G,D249Y^ switched the substrate stereoselectivity to accept the substrate enantiomer d-Arg with ∼6% relative activity. This study provides insights into the enzyme engineering of α-KG-dependent non-heme iron oxygenases at a molecular level and for the development of biocatalysts.

## Data Availability

The original contributions presented in the study are publicly available. This data can be found here: Protein Data Bank, 7VGL, 7VGN, 7Y5F, 7Y5I, 7Y5P, 7YHE, and 7YW9.
