# Supplementary material for: Crystal structure of the α-ketoglutarate-dependent non-heme iron oxygenase CmnC in capreomycin biosynthesis and its engineering to catalyze hydroxylation of the substrate enantiomer
Source: Front Chem. 2022 Sep 13;10:1001311. doi: 10.3389/fchem.2022.1001311 (PMC9513391; doi:10.3389/fchem.2022.1001311)
Supplement: Supplementary file 1 [file DataSheet1.docx]

# *Supplementary Materials*

**^1^H NMR spectrum for the CmnC product, compound 1**

**^13^C NMR spectrum for the CmnC product, compound 1**

**
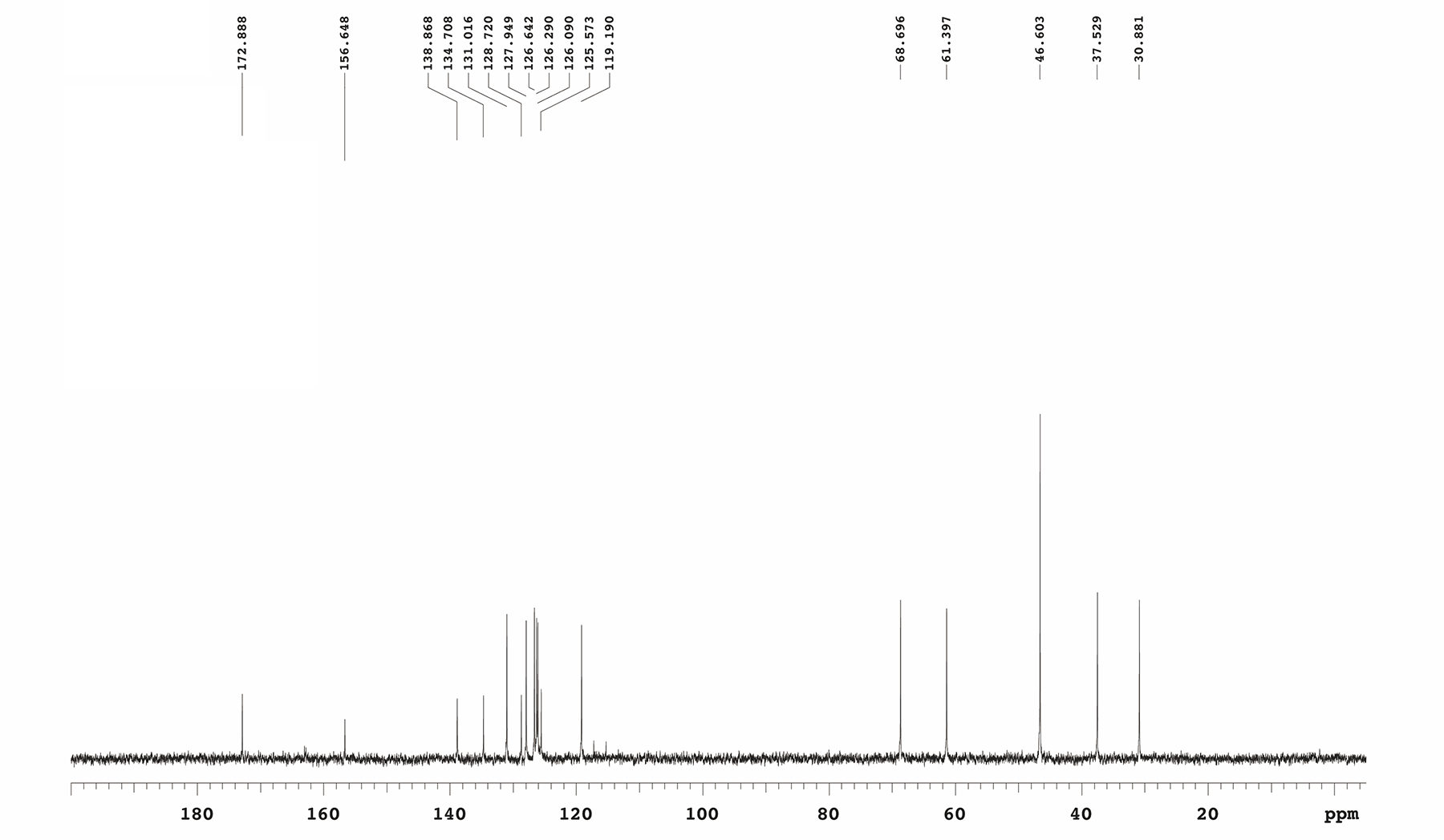
**

**^1^H COSY spectrum for the CmnC product, compound 1**

**
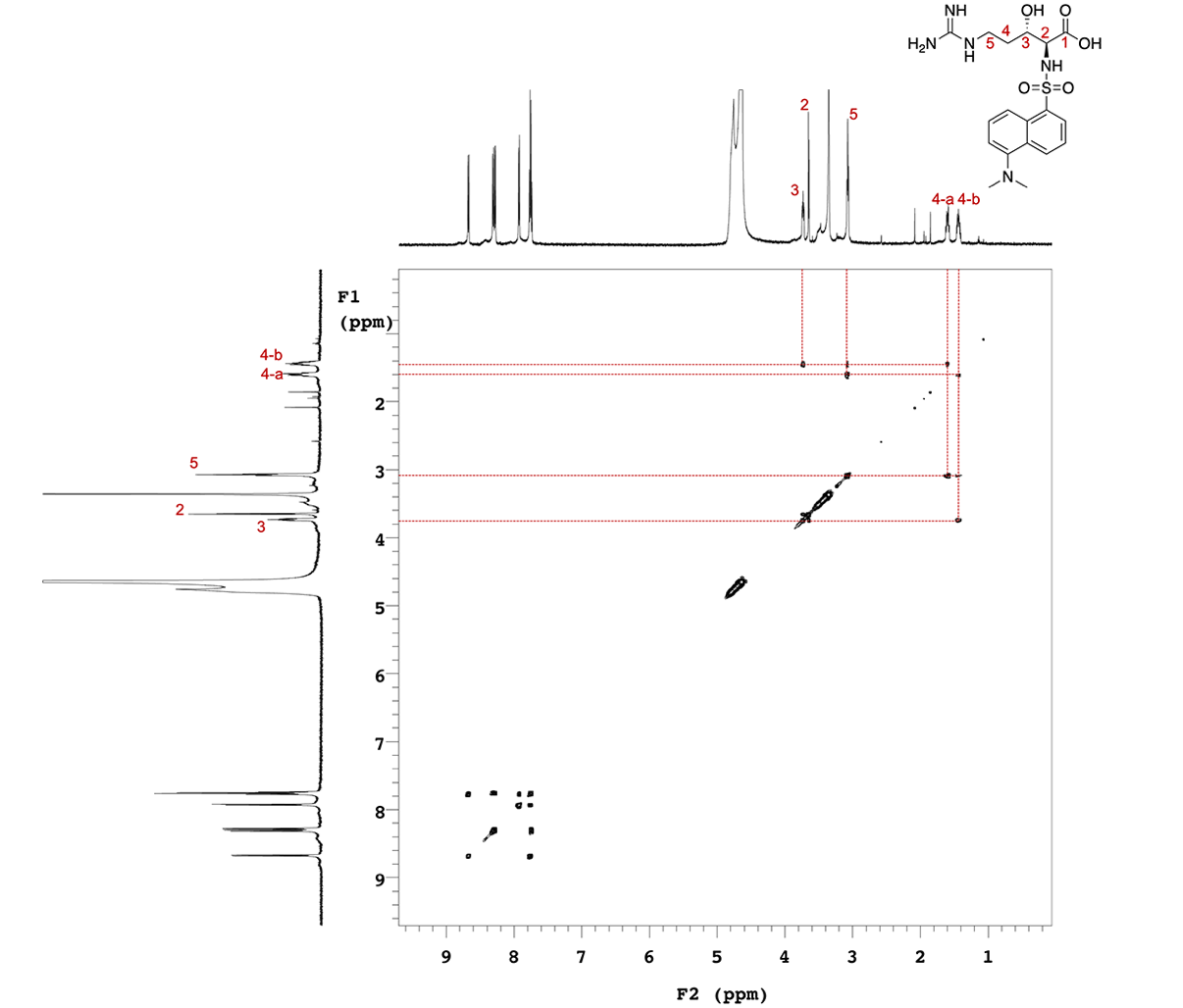
**

**^1^H ^13^C HSQC spectrum for the CmnC product, compound 1**

**
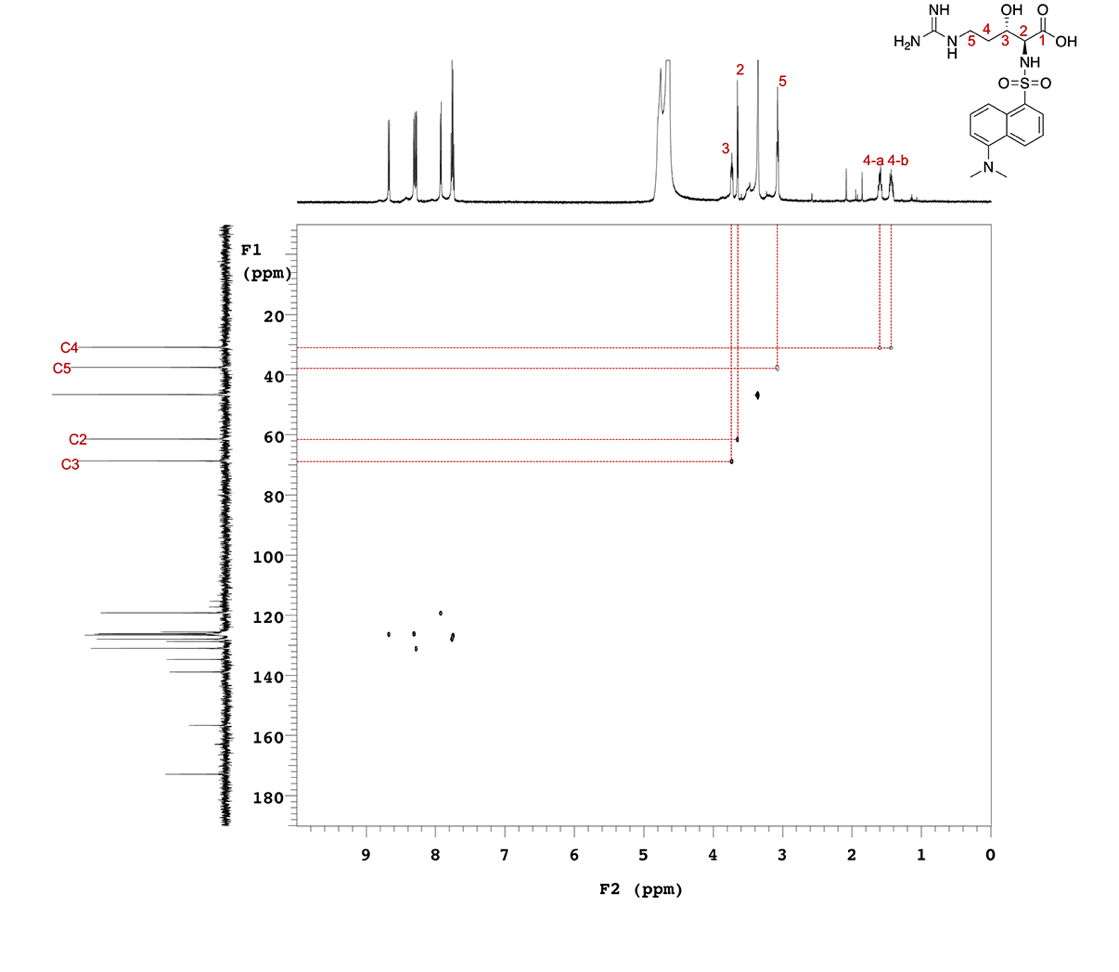
**

**Supplementary Figure S1.** NMR information for compound **1**. NMR spectra include ^1^H, ^13^C, COSY, and HSQC.

**
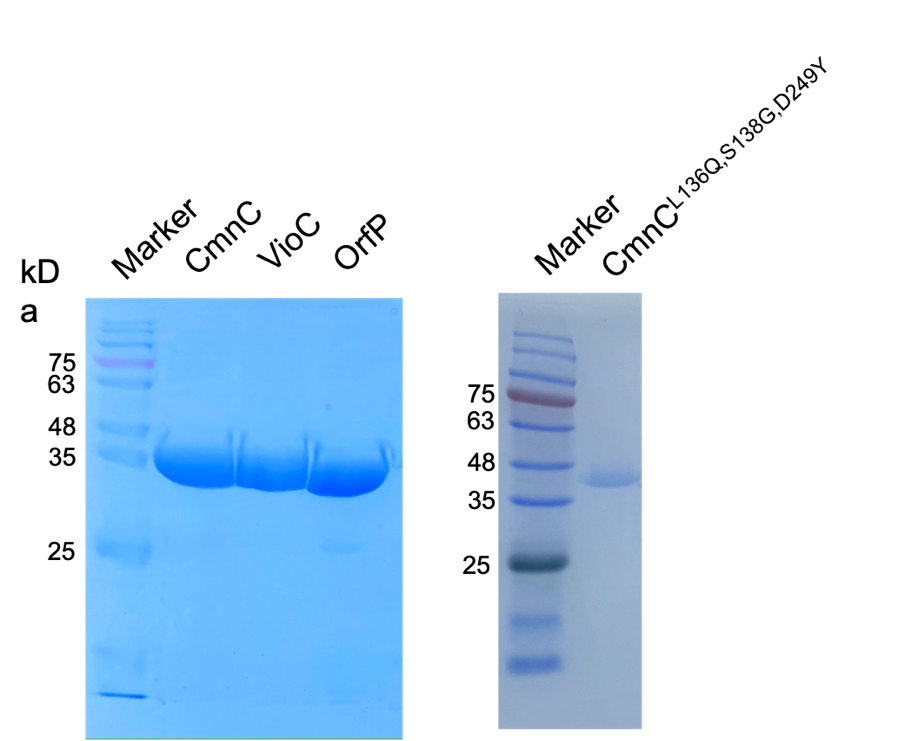
**

**Supplementary Figure S2.** SDS-PAGE of the enzymes in this study. The calculated molecular weight of CmnC, VioC, and OrfP are 39.03, 41.59, and 41.12 kDa, respectively.

**
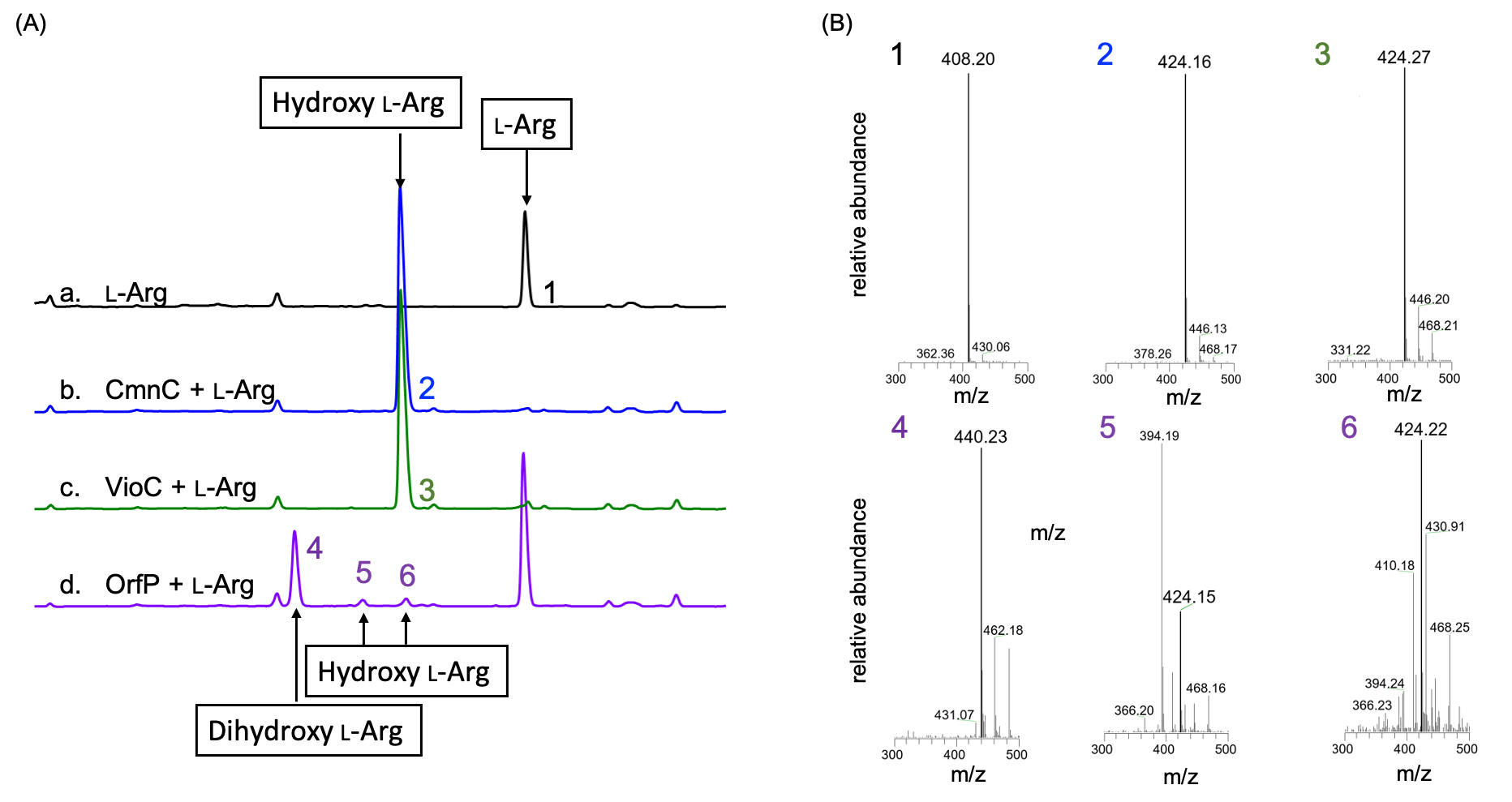
**

**Supplementary Figure S3.** LC-MS analysis of the CmnC, VioC, and OrfP with l-Arg as the substrate. (A) HPLC traces of the substrate l-Arg (a) and reactions in the presence of (b) CmnC, (c) VioC, and (d) OrfP with l-Arg. (B) Mass spectrometric analysis of the reaction products in (A). All samples were dansylated before LC-MS analysis.

**
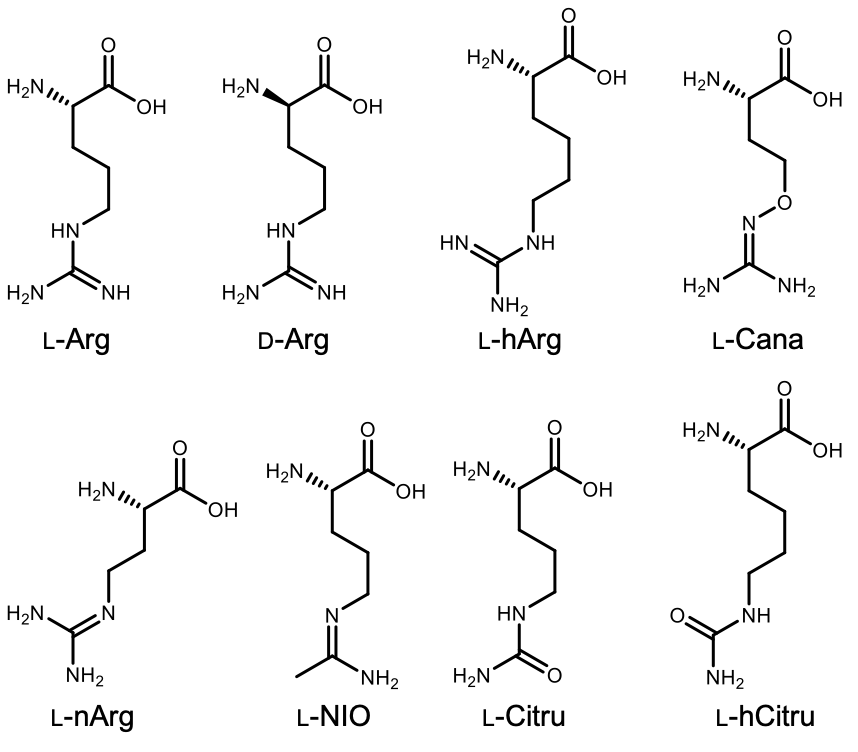
**

**Supplementary Figure S4.** l-Arg analogs used as substrates in this study.

**
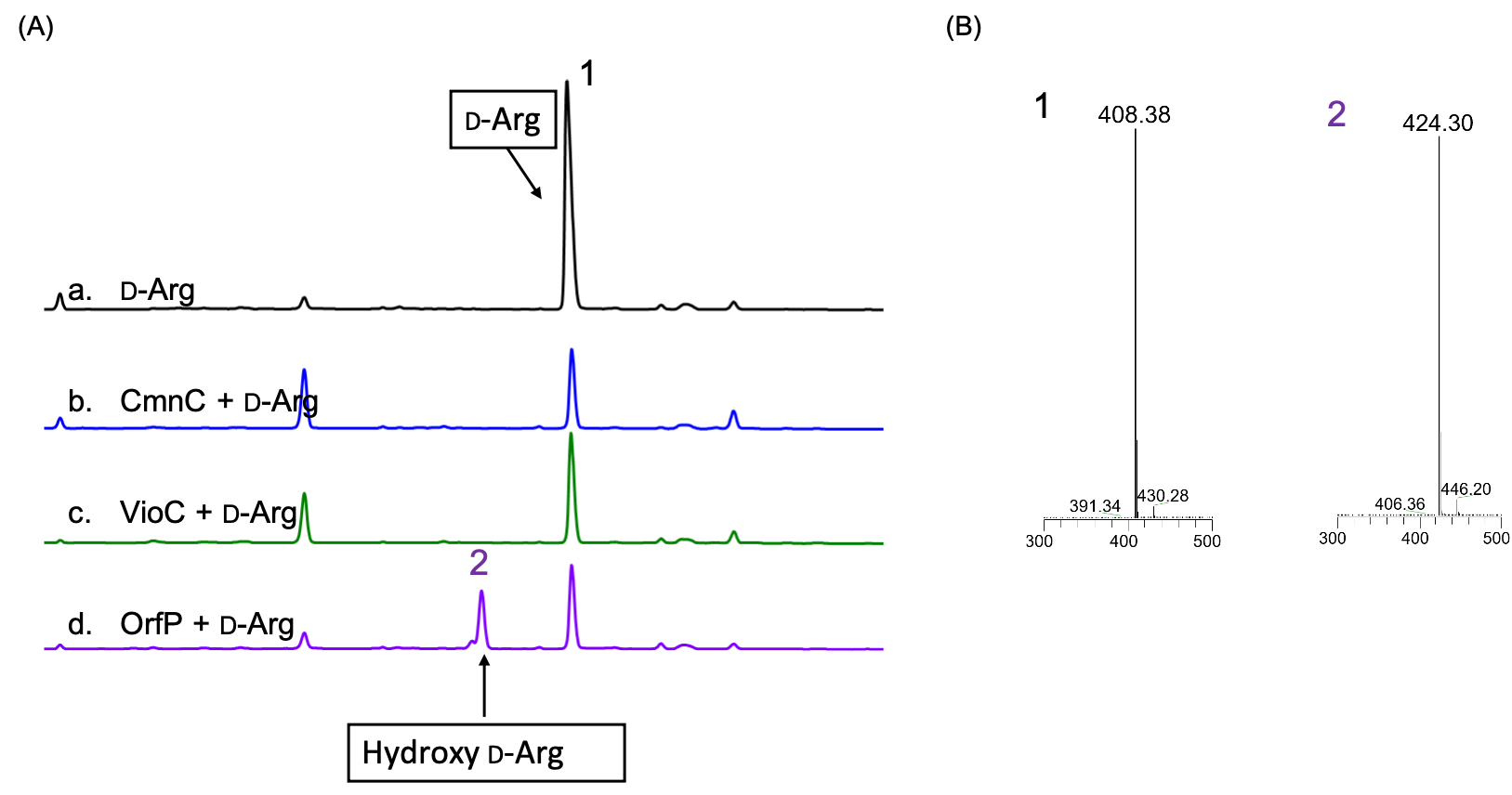
**

**Supplementary Figure S5.** LC-MS analysis of the CmnC, VioC, and OrfP with d-Arg as the substrate. (A) HPLC traces of the substrate d-Arg (a) and reactions in the presence of (b) CmnC, (c) VioC, and (d) OrfP with d-Arg. (B) Mass spectrometric analysis of the reaction products in (A). All samples were dansylated before LC-MS analysis.

**
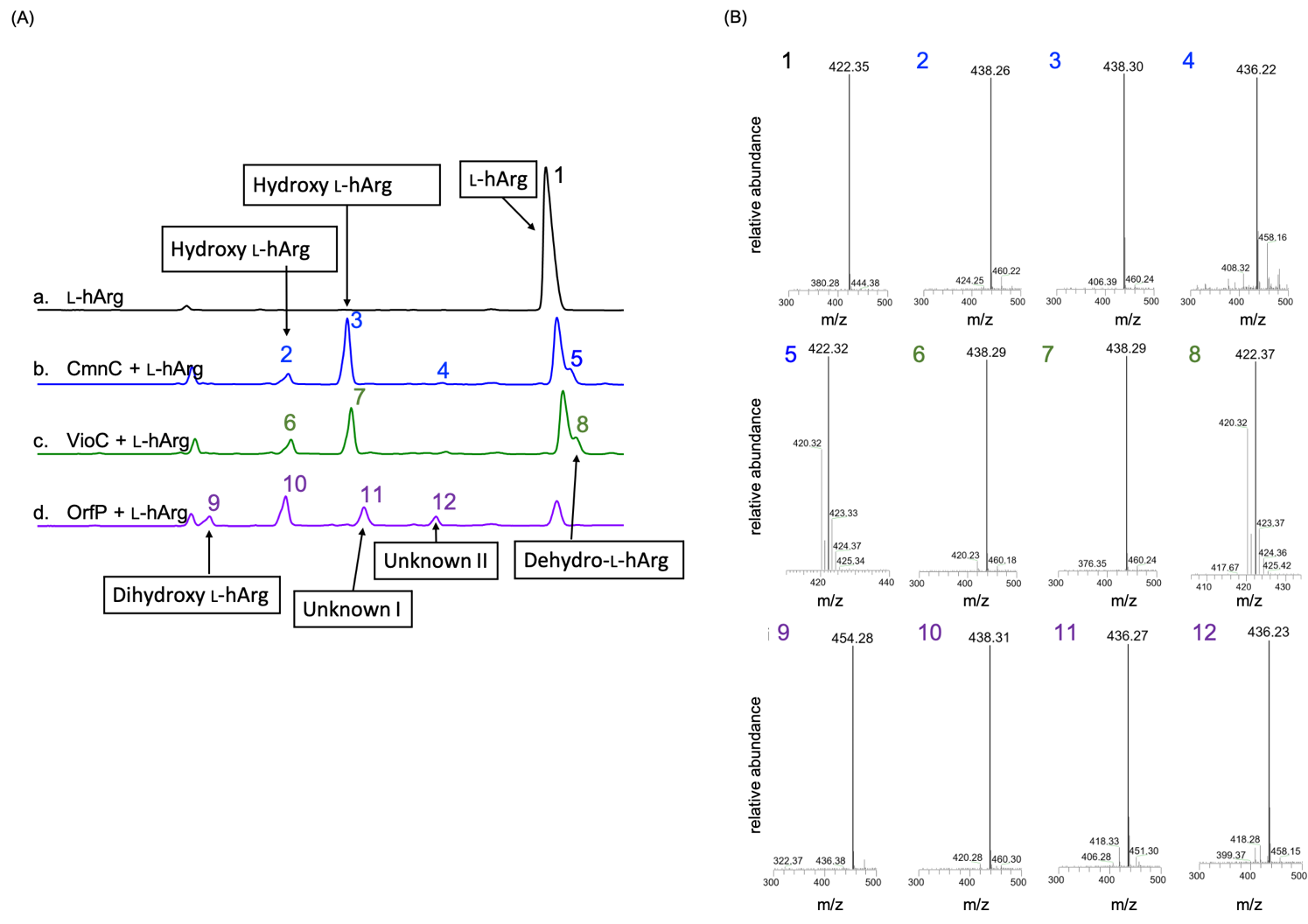
**

**Supplementary Figure S6.** LC-MS analysis of the CmnC, VioC, and OrfP with l-hArg as the substrate. (A) HPLC traces of the substrate l-hArg (a) and reactions in the presence of (b) CmnC, (c) VioC, and (d) OrfP with l-hArg. (B) Mass spectrometric analysis of the reaction products in (A). All samples were dansylated before LC-MS analysis.

**
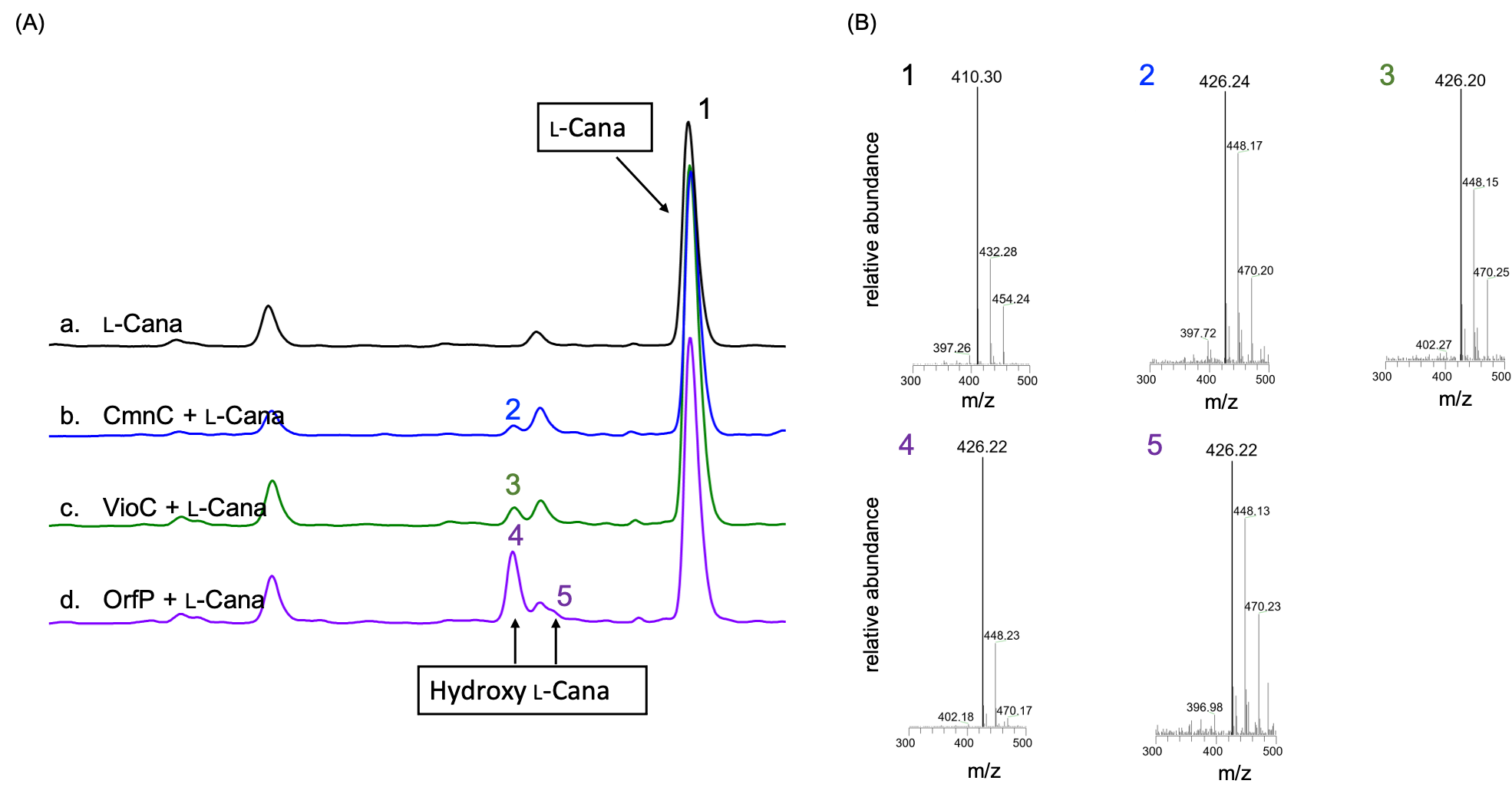
**

**Supplementary Figure S7.** LC-MS analysis of the CmnC, VioC, and OrfP with l-Cana as the substrate. (A) HPLC traces of the substrate l-Cana (a) and reactions in the presence of (b) CmnC, (c) VioC, and (d) OrfP with l-Cana. (B) Mass spectrometric analysis of the reaction products in (A). All samples were dansylated before LC-MS analysis.

**
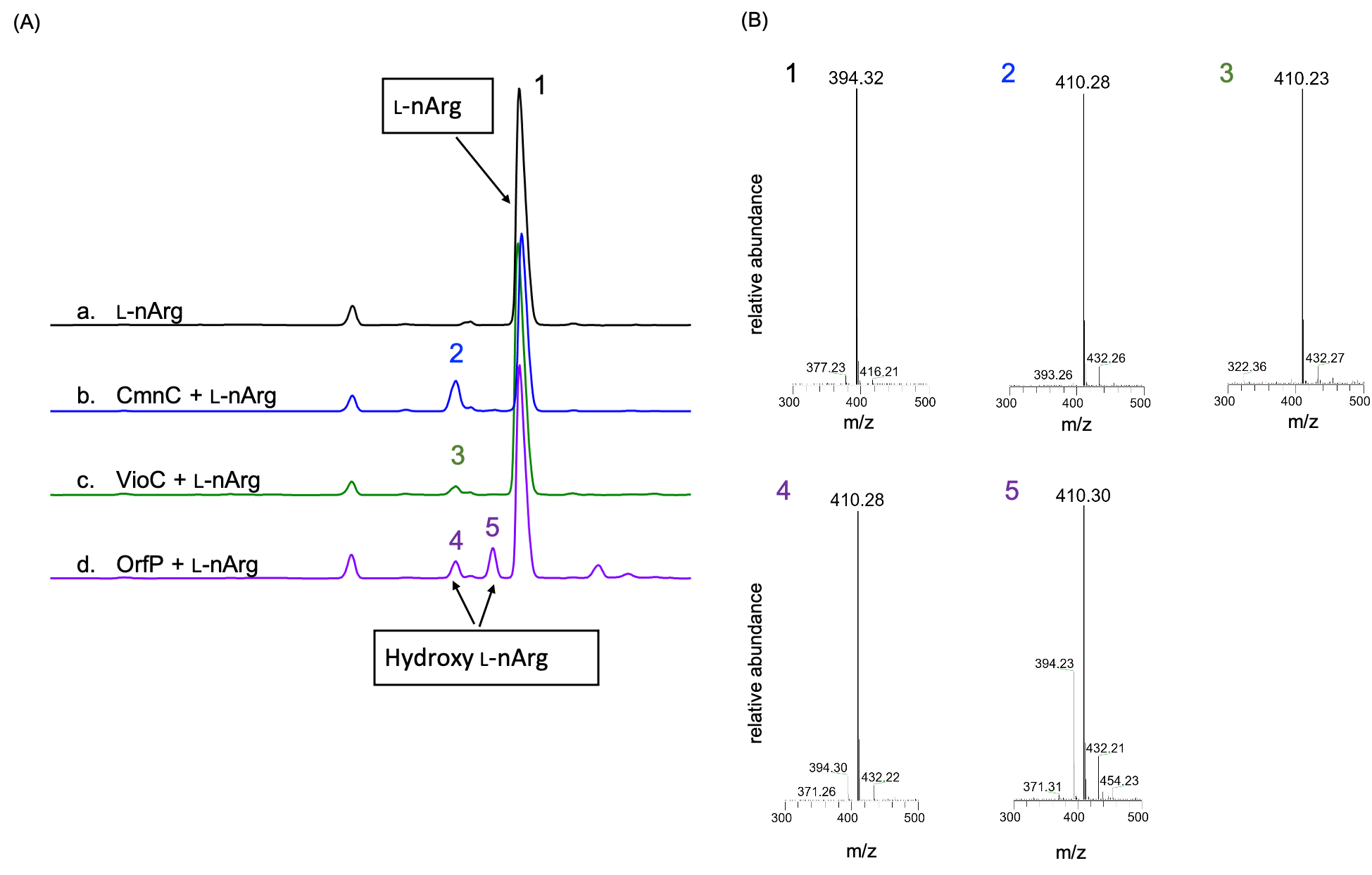
**

**Supplementary Figure S8.** LC-MS analysis of the CmnC, VioC, and OrfP with l-nArg as the substrate. (A) HPLC traces of the substrate l-nArg (a) and reactions in the presence of (b) CmnC, (c) VioC, and (d) OrfP with l-nArg. (B) Mass spectrometric analysis of the reaction products in (A). All samples were dansylated before LC-MS analysis.

**
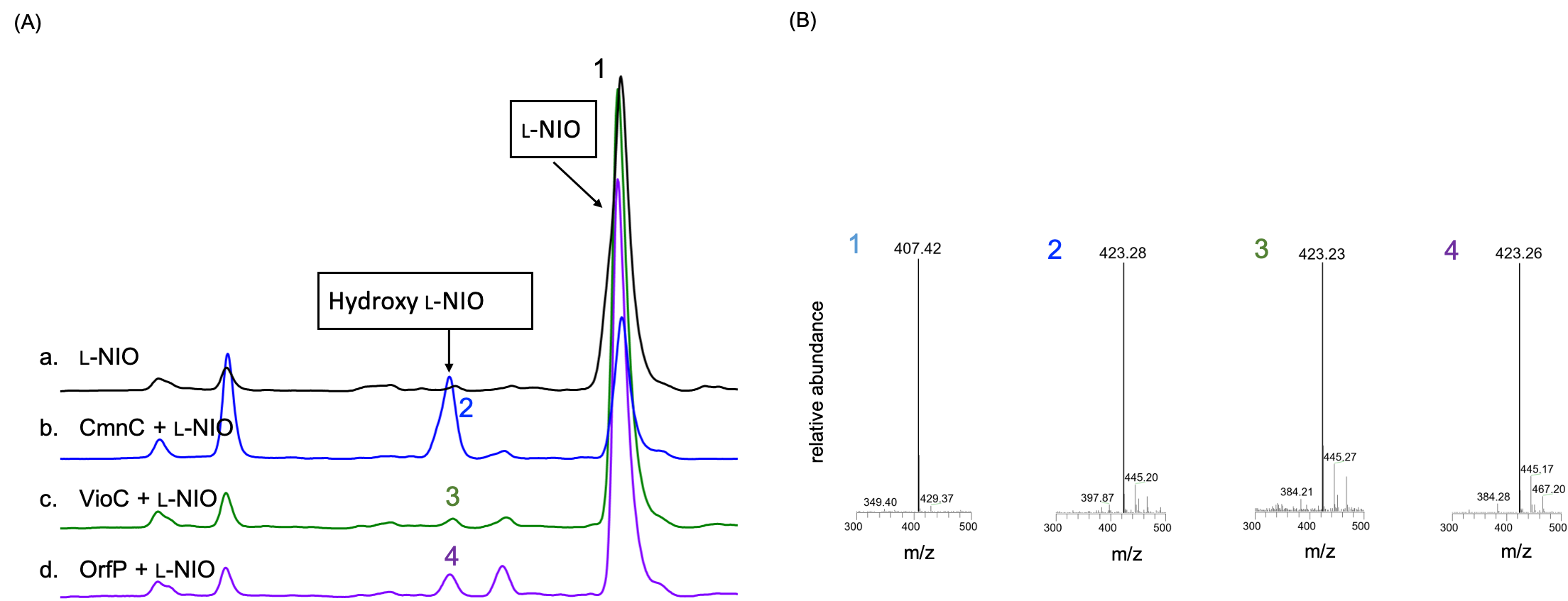
**

**Supplementary Figure S9.** LC-MS analysis of the CmnC, VioC, and OrfP with l-NIO as the substrate. (A) HPLC traces of the substrate l-NIO (a) and reactions in the presence of (b) CmnC, (c) VioC, and (d) OrfP with l-NIO. (B) Mass spectrometric analysis of the reaction products in (A). All samples were dansylated before LC-MS analysis.

**
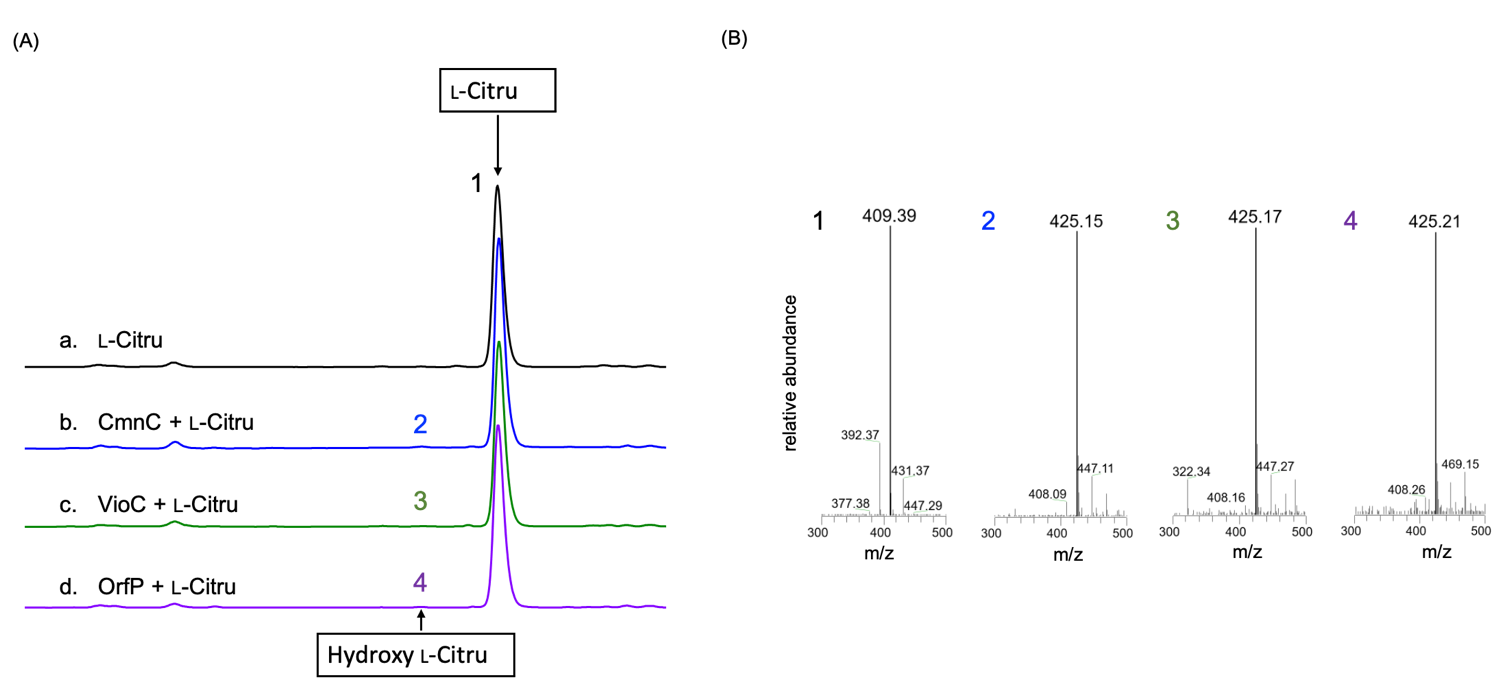
**

**Supplementary Figure S10.** LC-MS analysis of the CmnC, VioC, and OrfP with l-Citru as the substrate. (A) HPLC traces of the substrate l-Citru (a) and reactions in the presence of (b) CmnC, (c) VioC, and (d) OrfP with l-Citru. (B) Mass spectrometric analysis of the reaction products in (A). All samples were dansylated before LC-MS analysis.

**
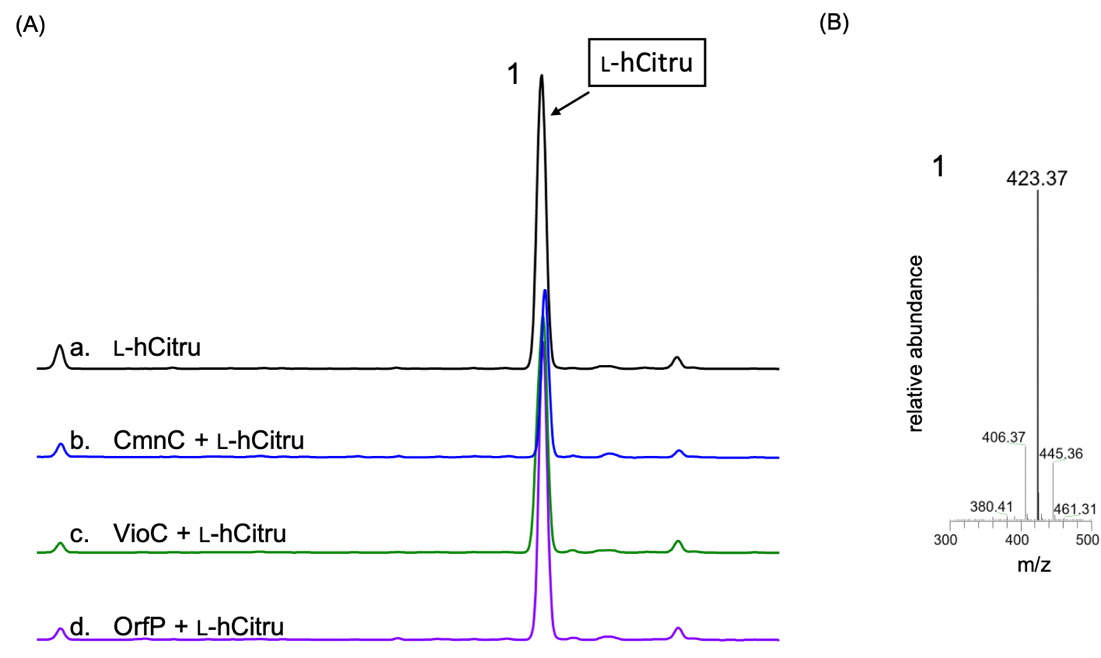
**

**Supplementary Figure S11.** LC-MS analysis of the CmnC, VioC, and OrfP with l-hCitru as the substrate. (A) HPLC traces of the substrate l-hCitru (a) and reactions in the presence of (b) CmnC, (c) VioC, and (d) OrfP with l-hCitru. (B) Mass spectrometric analysis of the reaction products in (A). All samples were dansylated before LC-MS analysis.

**
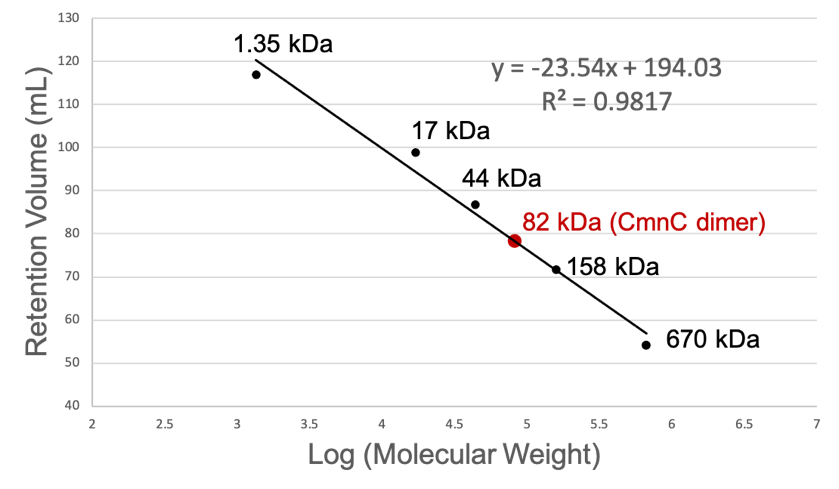
**

**Supplementary Figure S12.** Molecular weight estimation of CmnC by size exclusion chromatography. The calculated molecular weight of CmnC is 39.03 kDa. The apparent molecule weight of CmnC is estimated to be 82 kDa and suggesting that CmnC is a dimer.

**Supplementary Table S1.** Primers for CmnC and the triple mutant CmnC^L136Q,S138G,D249Y^ constructs

| hBH mutants | Primers |
| --- | --- |
| CmnC | Forward: 5’– GAACCCATATGACCGCCATCCGCGAGA –3’  Reverse: 5’– GAACCAAGCTTCACGCCTCACCCAGCA –3’ |
| CmnC^L136Q,S138G,D249Y^ | For L136Q and S138G:  Forward: 5’– TCGCAGGTCGGCTCCAGC –3’  Reverse: 5’– GCTGCTGGAGCCGACCTG –3’  For D249Y:  Forward: 5’– TGCGTGGACAGCTACTTCACCACC –3’  Reverse: 5’– GGCGGTGGTGAAGTAGCTGTCCAC –3’ |

**Supplementary Table S2.** Data collection, phasing, and refinement statistics for protein structures
